# 

**Published:** 1860-06

**Authors:** 


CONTENTS

OF THE

### Chicago Medical Journal for June, 1860.

ORIGINAL COMMUNICATIONS. PAGE.

ARTICLE 1.—Fracture of the Carpal Extremity of the Ulna. By James H. Farris, M. D.... 313
ARTICLE 2.—On the Sulphate of Iron. By R. C. Hamill, M. D.... 318
ARTICLE 3.—Case of Spinal Irritation. By C. D. Henton, M. D.... 324
ARTICLE 4.—Essay on Erectile Tumors. By Daniel Brainard, M. D.... 325
Report of the Chicago Charitable Eye and Ear Infirmary.... 356
Care of the Sick.... 358

EDITORIAL.

Book and Pamphlet Notices....359, 360
Tenth Annual Meeting of the Illinois State Medical Society.... 360
The Epidemic among Cattle.... 373

SELECTIONS.

Florence Nightingale.... 374
Opium an Antidote for Belladonna.... 375
Changes in Medical Schools.... 376
Ointment for Warts.... 376
South Carolina Medical College.... 376

